# Nanoscale temperature sensing of electronic devices with calibrated scanning thermal microscopy†

**DOI:** 10.1039/d3nr00343d

**Published:** 2023-03-23

**Authors:** Timm Swoboda, Nicolás Wainstein, Sanchit Deshmukh, Çağıl Köroğlu, Xing Gao, Mario Lanza, Hans Hilgenkamp, Eric Pop, Eilam Yalon, Miguel Muñoz Rojo

**Affiliations:** a Department of Thermal and Fluid Engineering, Faculty of Engineering Technology, University of Twente Enschede The Netherlands m.m.rojo@csic.es; b Faculty of Electrical Engineering, Technion – Israel Institute of Technology Haifa Israel; c Department of Electrical Engineering, Stanford University Stanford USA; d Faculty of Science and Technology and MESA+ Institute for Nanotechnology, University of Twente Enschede The Netherlands; e Materials Science and Engineering Program Physical Science and Engineering Division King Abdullah University of Science and Technology (KAUST) Thuwal Saudi Arabia; f Instituto de Micro y Nanotecnología, IMN-CNM, CSIC (CEI UAM+CSIC) Madrid Spain

## Abstract

Heat dissipation threatens the performance and lifetime of many electronic devices. As the size of devices shrinks to the nanoscale, we require spatially and thermally resolved thermometry to observe their fine thermal features. Scanning thermal microscopy (SThM) has proven to be a versatile measurement tool for characterizing the temperature at the surface of devices with nanoscale resolution. SThM can obtain qualitative thermal maps of a device using an operating principle based on a heat exchange process between a thermo-sensitive probe and the sample surface. However, the quantification of these thermal features is one of the most challenging parts of this technique. Developing reliable calibration approaches for SThM is therefore an essential aspect to accurately determine the temperature at the surface of a sample or device. In this work, we calibrate a thermo-resistive SThM probe using heater-thermometer metal lines with different widths (50 nm to 750 nm), which mimic variable probe-sample thermal exchange processes. The sensitivity of the SThM probe when scanning the metal lines is also evaluated under different probe and line temperatures. Our results reveal that the calibration factor depends on the probe measuring conditions and on the size of the surface heating features. This approach is validated by mapping the temperature profile of a phase change electronic device. Our analysis provides new insights on how to convert the thermo-resistive SThM probe signal to the scanned device temperature more accurately.

## Abstract 2

Nanoscale thermal sensing of devices with calibrated scanning thermal microscopy. The calibration allows converting the electrical response of thermo-resistive probes into temperature and considers variable probe-sample thermal exchange processes.

## Graphical abstract

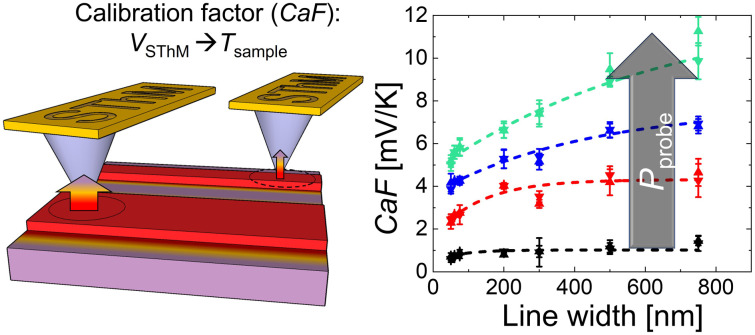

## Introduction

Scanning thermal microscopy (SThM) has become a popular scanning probe technique to measure nano- and micro-scale sample thermal features.^1–3^ SThM can be used to determine the thermal properties of different types of nanostructured materials, like polymeric nanowires,^4^ thermoelectric materials^5,6^ and phase change materials (PCM),^7^ when using thermal probes as small heaters and with proper calibration. Furthermore, calibrated SThM thermal probes can be also used as nanoscale sensors to obtain surface temperature maps. Compared to other spatially resolved thermometry techniques, such as infrared or Raman thermometry, SThM has the advantage of having a better spatial resolution.^8,9^ More recently, nanoscale spatially resolved temperature sensing approaches *via* SThM have attracted particular interest for determining the energy dissipated in electronic devices, where often heat hinders performance and reliability.^10^ For example, SThM has recently been applied to determine temperatures of transistors based on two-dimensional materials like MoS_2_,^11^ and memory devices like resistive random-access memory (RRAM)^12–14^ and phase change memory (PCM),^15^ as well as thermally-activated phase change devices based on VO_2_.^16^ The evaluation of the heat dissipated in individual electronic devices can open doors to establish new device engineering designs and architectures on the basis of devices’ thermal signatures.^17,18^ However, while SThM is a promising technique for the thermal characterization of electronic devices, its main challenge relates to its complex calibration. In this work, we use a thermo-resistive SThM probe, whose electrical resistance varies with temperature, to scan surface heater metal lines with multiple widths and applied power levels. Based on these measurements, we can determine how the SThM probe calibration factor, a parameter that converts electrical probe signals into temperature, varies under different measuring conditions.

SThM uses a temperature-sensitive probe, like a thermocouple,^19^ thermal expansion^20^ or a thermo-resistive probe.^1^ Among them, thermo-resistive probes are the most widely used for temperature sensing. During measurements, a small current is applied across the thermo-resistive element. This allows to track changes in the electrical resistance of the probe, which depends on temperature^21^ as described by

1 *R*_probe_(*T*) = *R*_0_·[1 + TCR·(*T* − *T*_0_)]

The probe electrical resistance (*R*_probe_) at temperature *T* can be calculated by means of a resistance reference value (*R*_0_) at temperature *T*_0_. The temperature coefficient of resistance (TCR), which is an intrinsic material specific property, defines the slope of the relation between resistance and temperature which in practice is usually linear. As a consequence, an increment of the temperature of the tip correlates with changes in the electrical resistance of the probe, and *vice versa*.^1,22,23^ Using this working principle, SThM can be used to obtain surface thermal maps with high thermal and spatial resolution (less than 1 K and ∼50 nm, respectively).^12,13^ However, the probe requires careful calibration to quantitatively correlate changes in the electrical resistance of the probe (mV) with temperature variations (K), *i.e.*, a calibration factor (*CaF*). For that purpose, several calibration approaches have been suggested in the past.

As an example, one common method for SThM calibration is based on measuring the electrical resistance of the probe while keeping it in contact with a hot-plate stage with an adjustable temperature.^24,25^ Alternatively, calibration approaches based on knowing the melting temperature of materials have also been used for thermo-resistive probes.^26^ In this approach, the probe is brought into contact with a material of well-defined melting point. The probe is heated until the material melts, which is detected by a sharp decrease in the probe deflection. With this method, the tip resistance can be correlated to the melting temperature of the sample under study. These methods are straightforward for application. However, they do not account for variations in the probe thermal exchange area and in the thermal sensitivity depending on the power applied to the thermo-resistive probe, which is especially relevant when scanning nanoscale heating features.

More recently, Deshmukh *et al.*^13^ employed nanoscale metal lines to determine a *CaF* that transforms the electrical SThM probe response into temperature changes.^13^ They observed a change in the *CaF* depending on the width of the heating metal lines, which was correlated to variations in the tip–sample thermal exchange radius. Since the SThM measurements were made in-contact, this approach used an electrical insulating capping layer between the tip and the sample that avoids conducting surfaces to interfere with the electrical signal of the Wheatstone bridge or even probe damage. This feature is especially relevant for the characterization of electronic devices when sensitive thermo-resistive probes in contact mode are used. Additionally, it allows comparability of the results with samples of similar capping surfaces, *i.e.*, comparable thermal contact resistance between tip and sample. If the sample cannot be coated, alternatives such as SThM measurements in non-contact mode^27^ or depositing an insulating capping layer to the thermo-resistive tip could be a possibility. To advance on the calibration approach presented in ref.^13^ it is essential to study the influence of a broader range of line widths as well as the impact of the self-heating probe to better understand their influence on the *CaF*.

In this work, we extend the results of the calibration method described in reference.^13^ We use palladium (Pd) on silicon nitride (SiN) based thermo-resistive SThM probes^22^ to characterize the heating produced by thin Pd metal lines of different widths. Pd possesses a high and well-known TCR, which makes it an ideal material to use in this experiment, to characterize and to compare with previous results.^28–30^ We carefully evaluate the *CaF* based on the probe-sample thermal exchange area, which causes different line widths to yield different SThM probe temperatures. Additionally, we investigate the impact of the power applied to the SThM probe to sense temperature at the surface. Apart from that we characterize the heating behaviour for each power applied to the probe. Overall, we aim to shed light on the need to carefully choose the *CaF* based on the size of the sample as well as the tip–sample energy balance.

## Experimental setup

**Fig. 1:**
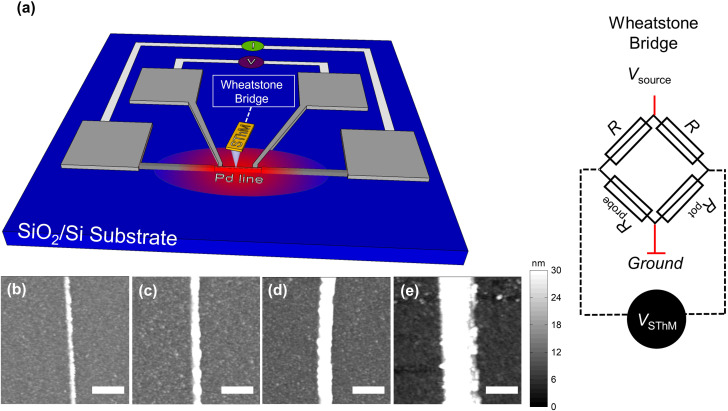
(a) Sample and measurement configuration. Palladium (Pd) lines with different widths (50–750 nm) and four Pd pads are deposited on SiO_2_/Si substrate. For the measurement, the heated lines are scanned using SThM. A Wheatstone bridge is used to track changes in the electrical resistance of the probe and consists of four resistors: two having fixed resistances *R*, a potentiometer *R*_pot_ and the resistance of the probe *R*_probe_. A voltage with variable magnitude is applied between *V*_source_ and the ground during the measurements. (b–e) AFM topography images of different Pd lines at widths of (b) 50, (c) 75, (d) 200, (e) 500 nm, scanned at tapping mode. (scale bar equal to (b) 400 nm, (c) 600 nm, (d) 700 nm, (e) 700 nm).

The measurement approach of our calibration, including the SThM setup and the calibration sample structure, is sketched in Fig. 1(a). Regarding our calibration samples, we used Pd metal lines of different widths (50–750 nm) deposited on a SiO_2_/Si substrate. We patterned and deposited the metal heating lines by e-beam lithography and e-beam evaporation (see ESI S1† for details). We capped the devices with a thin layer of Al_2_O_3_ to keep the sample electrically isolated from the SThM probe (see ESI S1† for details). Fig. 1(b–e) show the topography of metal lines with different widths that were obtained using an atomic force microscope (AFM) in tapping mode (see ESI S2† for details). First, the metal lines were characterized electrically to determine their electrical resistance. For that purpose, we used four-point probe measurements as shown in Fig. 1(a). We applied current between the two exterior pads while measuring the potential difference across the inner ones. Using this approach, the resistance of the line was measured as a function of the applied power. Since the resistance of a thermo-resistive Pd line depends on its temperature linearly,^31^ as described by eqn (1), one observes a linear increase of the resistance with the power applied. Afterwards, we characterized the electrical resistance of the lines at different stage temperatures. Based on these measurements, we were able to determine the resistance at zero power, *i.e. R*_0_, for each temperature of the stage (*T*_stage_). By plotting *R*_0_*vs*. the temperature of the stage we calculated the TCR of each line from the slope of the *R*_0_*vs. T*_stage_ graph. In combination with the resistance *vs*. power data, we extracted the temperature increase of the lines as a function of the power applied to them (see ESI S3† for details).

**Fig. 2:**
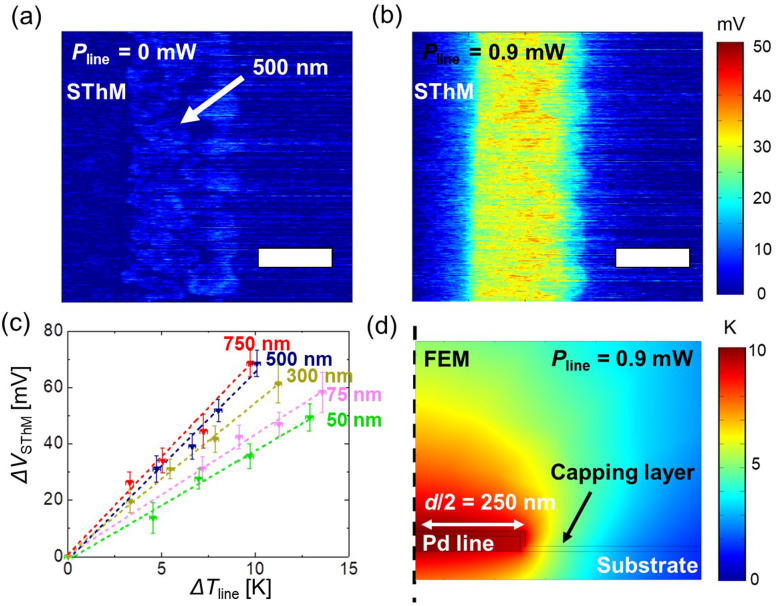
(a and b) SThM maps of a 500 nm wide line at (a) 0 W, (b) 0.9 mW power applied, scanned while a voltage of 0.5 V is applied at the Wheatstone bridge (scale bar equal to 500 nm). Variations observed at the line in the non-heated case are originated by differences in the tip–surface interaction. (c) Changes of the SThM signal measured at a heated line as a function of the temperature increase respect to the non-heated case for different widths. (d) FEM results for the heating of a 500 nm wide line at an applied power of 0.9 mW. The dashed line at the left edge indicates the plane of symmetry.

In operation we apply a voltage across the Wheatstone bridge to induce a small current that allows us to monitor changes in the electrical resistance of the probe. Given the thermo-resistive nature of our probes an increase in the probe temperature results in an increment of the resistance of the probe.^1^ In contact with the surface, we adjust *R*_pot_ to be equal to *R*_probe_, balancing the bridge. In this configuration, the nullified bridge voltage, which we refer to as SThM signal *V*_SThM_ (shown in Fig. 1(a)), is approximately proportional to the change in *R*_probe_ and allows changes in probe temperature to be sensed as the probe scans over the sample. Fig. 2(a) shows a flattened 2D SThM plot obtained when scanning a non-heated metal line with a width of 500 nm. As expected the SThM signal remained constant along the scan, with minor topography related differences at the line as consequence of tip–surface interaction changes.^32^ In Fig. 2(b) we show a flattened SThM map of the same line when heating the line by applying an electrical power *P*_line_ of 0.9 mW. In this case we observed a significant increment of the SThM signal at the location of the heated metal line. For the purpose of a better illustration, we applied a zero order flattening on the images in Fig. 2(a) and (b). For the characterization of the *CaF* we used the raw data later. We repeated the measurements while heating the lines at different powers. The magnitude of the signal linearly depends on the heating power and hence on the temperature rise of the line. Fig. 2(c) shows the rise in the SThM signal observed at the line (Δ*V*_SThM,line_) plotted against the corresponding temperature increase of the line during the measurements (Δ*T*_line_) for various line widths. Δ*T*_line_ was obtained from the four-point probe measurements. To subtract the influence of the topography on our results, we determined Δ*V*_SThM,line_ at Δ*T*_line_ as the difference of the maximum SThM signal in the heated case *V*_SThM,max,line_(Δ*T*_line_) *vs*. the maximum SThM signal at the non-heated *V*_SThM_,_max,line_(0) case as follows:2
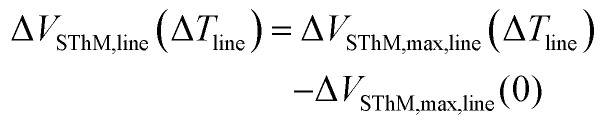


From the graphs in Fig. 2(c) we extracted our calibration factor (*CaF*) as the slope of the Δ*V*_SThM,line_*vs*. Δ*T*_line_ graphs.

## Results

Fig. 2(c) presents the Δ*V*_SThM,line_*vs*. Δ*T*_line_ behaviour, which increases until it saturates at higher line widths. This behaviour can be explained from the thermal exchange interaction between the tip and sample surface. The heat exchange between the tip and the sample is typically considered as a circular area with a defined thermal exchange radius.^23^ The intrinsic thermal exchange diameter around the tip is given by its heat transfer mechanisms (solid–solid, water meniscus, convection, and radiation). Then, when this thermal exchange radius is larger than the width of the line, heat exchange is reduced and the SThM signal drops. As a result, we observed a decrease of the *CaF* below a certain cut-off line width. These results match with previously obtained results.^13^

Additionally, we used a finite element model (FEM) to verify the temperature of the lines against the applied power, as shown in Fig. 2(d). Therefore, we replicated the sample configuration using the same thickness values and material properties as in the sample fabrication. In our model we correlated the temperature of the lines to the power values measured by the electrical characterization. The results obtained with the FEM agree well with the four-point measurements. Additionally, we observed that the calculated temperature drop between Pd line and surface on top of the capping layer is well below 1 K (see ESI S4† for details).

Since the heat exchange between the probe and the sample changes significantly with the voltage applied to the Wheatstone bridge *V*_source_, we further conducted measurements to estimate its impact on the calibration. The larger the power applied to the probe, the higher its temperature during the scan. Aiming to evaluate the impact of the probe heating on the *CaF*, we conducted the same Δ*V*_SThM,line_*vs*. Δ*T*_line_ analysis for four different Wheatstone bridge voltages (*V*_source_ = 0.1, 0.3, 0.5 and 0.7 V). However, *V*_source_ is difficult to compare between different probes as it depends on the resistance of the probe. Thus, we measured the total resistance *R*_probe,total_ = 340 Ω of the probe by measuring its IV behaviour. This total resistance includes the thermo-resistive element but also two current limiters (∼101.5 Ω each). After subtraction, the resistance of the thermo-resistive Pd element is *R*_probe_ = 137 Ω. Considering the resistances of the Wheatstone bridge, we then estimated the power applied to the probe during our measurements for each *V*_source_. For the four measuring configurations, we calculated the power values *P*_probe_ to be 0.8 μW, 7 μW, 19 μW and 37 μW during the measurements (see ESI S5† for details).

**Fig. 3:**
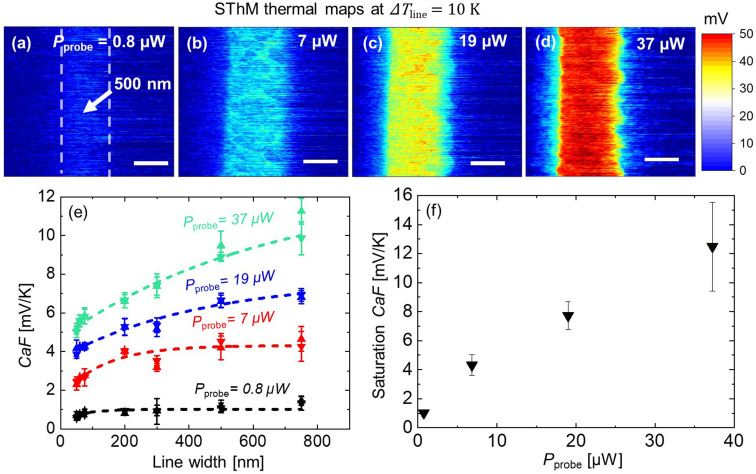
(a–d) SThM thermal maps of a 500 nm wide metal line at Δ*T*_line_ = 10 K when scanning at probe powers (*P*_probe_) of (a) 0.8 μW, (b) 7 μW, (c) 19 μW, (d) 37 μW (scale bar equal to 400 nm). (e) Calculated calibration factor (*CaF*) for different power values as a function of the line width of the scanned metal lines (upwards triangle correspond to the trace signal, downwards triangle correspond to the retrace signal). (f) Estimated saturated *CaF* as a function of *P*_probe_.

Fig. 3(a–d) show four different flattened SThM thermal maps of the same metal line width of 500 nm. The same power was applied to the line for the different SThM scans, achieving a Δ*T*_line_ of 10 K. However, we varied the power magnitude of *P*_probe_ as seen in Fig. 3(a-d) and stated in the figure caption, *i.e.*, from 0.8 to 37 μW. By comparing the four figures we observed that the contrast at the line increases with *P*_probe_. In other words, we see a clear contrast between the line signal and the substrate signal in Fig. 3(d), while the line signal is less distinguishable in Fig. 3(a). The same measurements were conducted for each line width and at four different powers (see ESI S6†) to determine the corresponding *CaF*.

At this point it is worth noting that we calculated *CaF* by means of the difference of the raw signal at the heated line and the raw signal of the line in a non-heated reference scan (see ESI S7†). By using this approach, we were able to plot the *CaF* as a function of the line width for all the four configurations described above in Fig. 3(e). We verified our results by conducting our measurements with a second probe (see ESI S8† for details). Furthermore, we observed that the *CaF* keeps on increasing when applying *P*_probe_ values beyond the configurations displayed here (see ESI S9† for details).

## Discussion and SThM application to measure electronic devices

Based on the results shown in Fig. 3, we observed an increase of the *CaF* as a function of *P*_probe_. The increment of *P*_probe_ is a result of pushing higher currents *I*_probe_ through the probe because of increasing *V*_source_. *I*_probe_ directly impacts the slope of the Δ*V*_SThM_*vs*. Δ*T*_probe_ (*V*_SThM_ = *I*_probe_·*R*_probe_) function. As a result of that we should expect a stronger increase in *V*_SThM_ for the same temperature increase when operating at a higher current. Apart from that applying more power to the tip results in a higher probe temperature. This is confirmed by the linear increase of the probe temperature with *P*_probe_ independent of the heating of the line (see ESI S10†). The increment of heating power and probe temperature causes an increase of the cut-off line width at which the *CaF* started to saturate at higher *P*_probe_, as can be seen in Fig. 3(e). The cut-off line width tells us the size at which the heat transport between probe and sample starts to truncate and is therefore an indicator of the thermal exchange area. As we increase the power applied to the tip, we consequently increase the thermal exchange area around the tip vicinity. We fitted the data in Fig. 3(e) graphs with a simple exponential function to estimate the saturation value of *CaF* as drawn by the dotted lines. For higher power values the fit saturates at line widths above 750 nm. Fig. 3(f) shows the saturation *CaF* values as a function of *P*_probe_, showing a steady trend.

This new information enlarges the toolkit of operating a calibrated SThM system. Depending on the needs of the measurement the bridge power can be adjusted. For example, one could choose to operate the SThM at a higher power to increase the temperature sensitivity. However, at lower power one obtains less self-heating of the probe and therefore could be the preferred option in other cases.

**Fig. 4:**
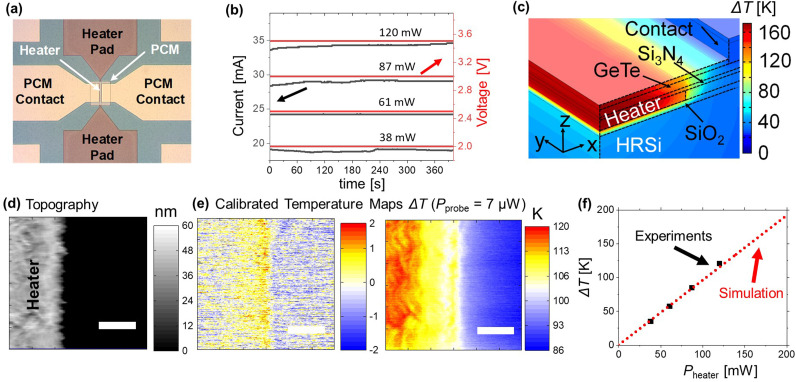
(a) Setup of the PCM sample. (b) Applied current and measured voltage as a function of the measurement time. (c) FEM simulation of the measured sample. (d) Topography of the investigated PCM sample obtained with a SThM probe. (e) Calibrated temperature maps of the PCM sample without and with power applied (120 mW) and for a power applied to the probe of *P*_probe_ = 7 μW (scale bar of (d) and (e) equal to 400 nm). (f) Calculated temperature increase at the line obtained with FEM as illustrated in (c) and from experiments, using the *CaF* obtained in Fig. 3.

Finally, to verify the results of the calibration, we assessed the *CaF* in electronic devices. We investigated the heating characteristics of a phase change material (PCM) device which we characterized in a previous study.^33^Fig. 4(a) shows the sample schematics revealing the PCM sputtered on a 1.5 μm wide metal heater line. We capped the surface of the PCM sample with a thin insulation layer of SiO_2_ to electrically isolate the tip from the sample. We scanned the sample surface with our SThM probes while heating the metal line by applying an electrical current between the two heater pads. We repeated the measurements for four power configurations. The applied current and voltage at the metal lines as a function of the run time of the steady state measurements is shown in Fig. 4(b). The finite element model of the PCM structure was reported previously^33,34^ to determine the value of the temperature of the device based on the power applied. The model considers the thermal conductivity and capacitance of each layer, including the thermal boundary resistance of the interfaces as well as the temperature coefficient of resistance of the heater.

In this FEM, illustrated in Fig. 4(c), the structure of the device was replicated and a power source was applied to the heater. We calculated the surface temperature to compare them with the calibrated temperature maps obtained with SThM. As an example, the topography and converted temperature maps of one of the heater lines are presented in Fig. 4(d) through (f). Fig. 4(e) displays converted SThM temperature maps obtained with 0 W and 120 mW applied to the device during the scan, respectively. As expected, the maximum temperature is observed towards the centre of the heater line area. We converted the signal as described above by determining the difference of the raw SThM signal in the heated *vs*. the non-heated case. During the scan we applied a power of 7 μW to the probe. The line width significantly exceeded the cut-off value of this power configuration. Therefore, we used the saturation *CaF* (= 4.31 mV K^−1^) of this power configuration as shown in Fig. 3(e). We then calculated the expected temperature increase of the lines with four different power configurations (see Fig. 4(b)). Fig. 4(f) shows the maximum temperature increase of the heated area *vs*. the power applied to the lines. It can be observed that, the results obtained by the calibrated SThM (represented by the black squares) and the results of the FEM simulation illustrated by the red dotted line,^33^ are in good agreement. Hence, it can be concluded that this calibrated SThM approach is a promising technique to characterize the temperature of different samples and devices with nanoscale accuracy. A potential source of error might originate because of differences in the probe to sample contact between calibration sample and device due to capping layer. However, the capping layer of SiO_2_ and Al_2_O_3_ present similar surface roughness and thermal conductivities. Therefore, we estimate that this difference is bound to be less than 3%, which agrees well with the analysis of the temperature increase of the PCM sample.

## Conclusions

In conclusion, in this work we determined the calibration factor of a thermo-resistive SThM probe using a set of metal lines with different widths and powers applied to them. We determined that the calibration factor depends on the thermal exchange area between the tip and the sample as well as on the power applied to the probe. This calibration method enables adjustable SThM measurement to quantify the heating at the surface of a sample depending on the needed thermal sensitivity and local features of the heating surface. Therefore, the outcome of this work displays the advantages and disadvantages of operating the SThM at different bridge power values. For example, one could prefer to operate the SThM at a higher power to increase the thermal contrast of the measurements. On the other hand, one could operate at a lower power to avoid heating of the sample due to the probe. Moreover, the validation of the calibration results shows that this method can be applied for the characterization of similar structures. Therefore, the results showed the flexibility of the SThM to conduct temperature mapping for a wide range of materials and devices with nanoscale spatial resolution. This technique sheds light on how to carefully calibrate and use SThM for accurate surface thermal sensing. Future studies should put emphasis on investigating the impact of material specific properties such as the surface roughness, probe thermal contact resistance or thermal conductance on the thermal exchange between the probe and sample.

## Author contributions

Conceptualization, T.S. and M.M.R.; formal analysis, T.S., N.W., S.D. and Ç.K.; investigation, T.S., N.W. and X.G.; methodology, T.S. and M.M.R.; supervision, M.M.R., H.H., E.P., E.Y, M.L.; visualization, T.S. and M.M.R.; writing – original draft T.S. and M.M.R.; writing – review & editing, all authors have contributed to the review and editing process of the manuscript preparation.

## Conflicts of interest

There are no conflicts to declare.

## Supplementary Material

NR-015-D3NR00343D-s001

NR-015-D3NR00343D-s002
