# Supplementary material for: Nanoscale temperature sensing of electronic devices with calibrated scanning thermal microscopy
Source: Nanoscale. 2023 Mar 23;15(15):7139–46. doi: 10.1039/d3nr00343d (PMC10099078; doi:10.1039/d3nr00343d)
Supplement: NR-015-D3NR00343D-s001 [file NR-015-D3NR00343D-s001.pdf]

Electronic Supplementary Material (ESI) for Nanoscale.
This journal is © The Royal Society of Chemistry 2023

Figure S1

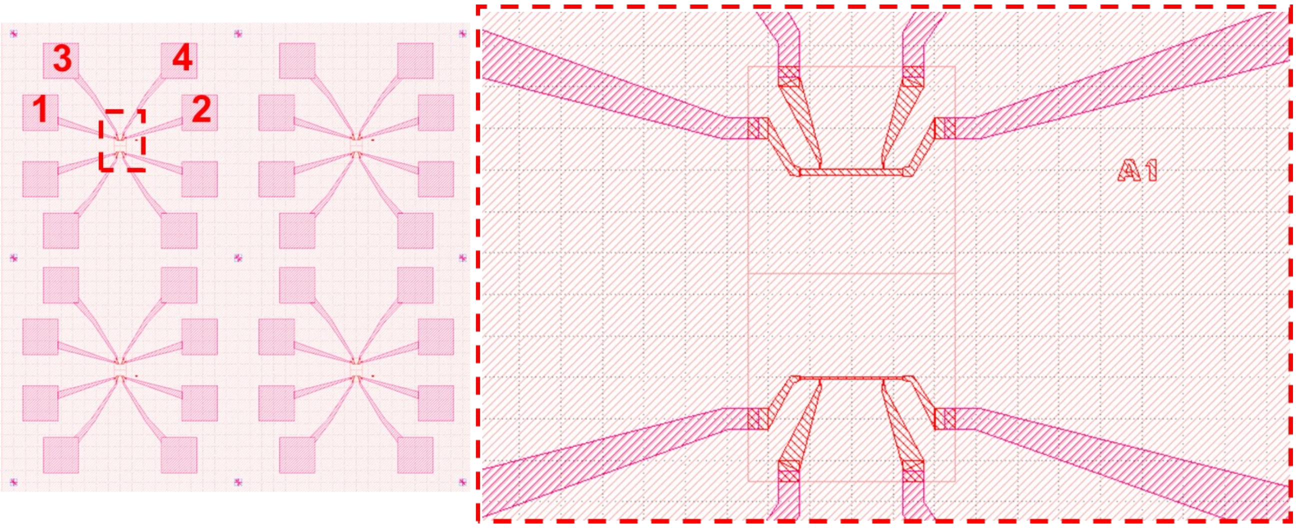

Figure S2

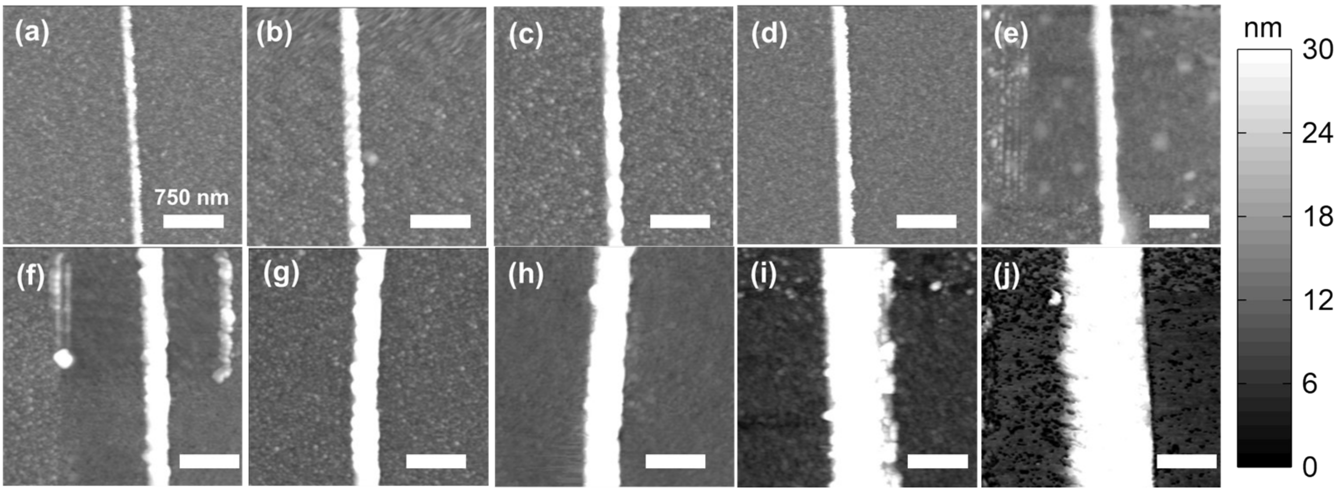

Figure S3

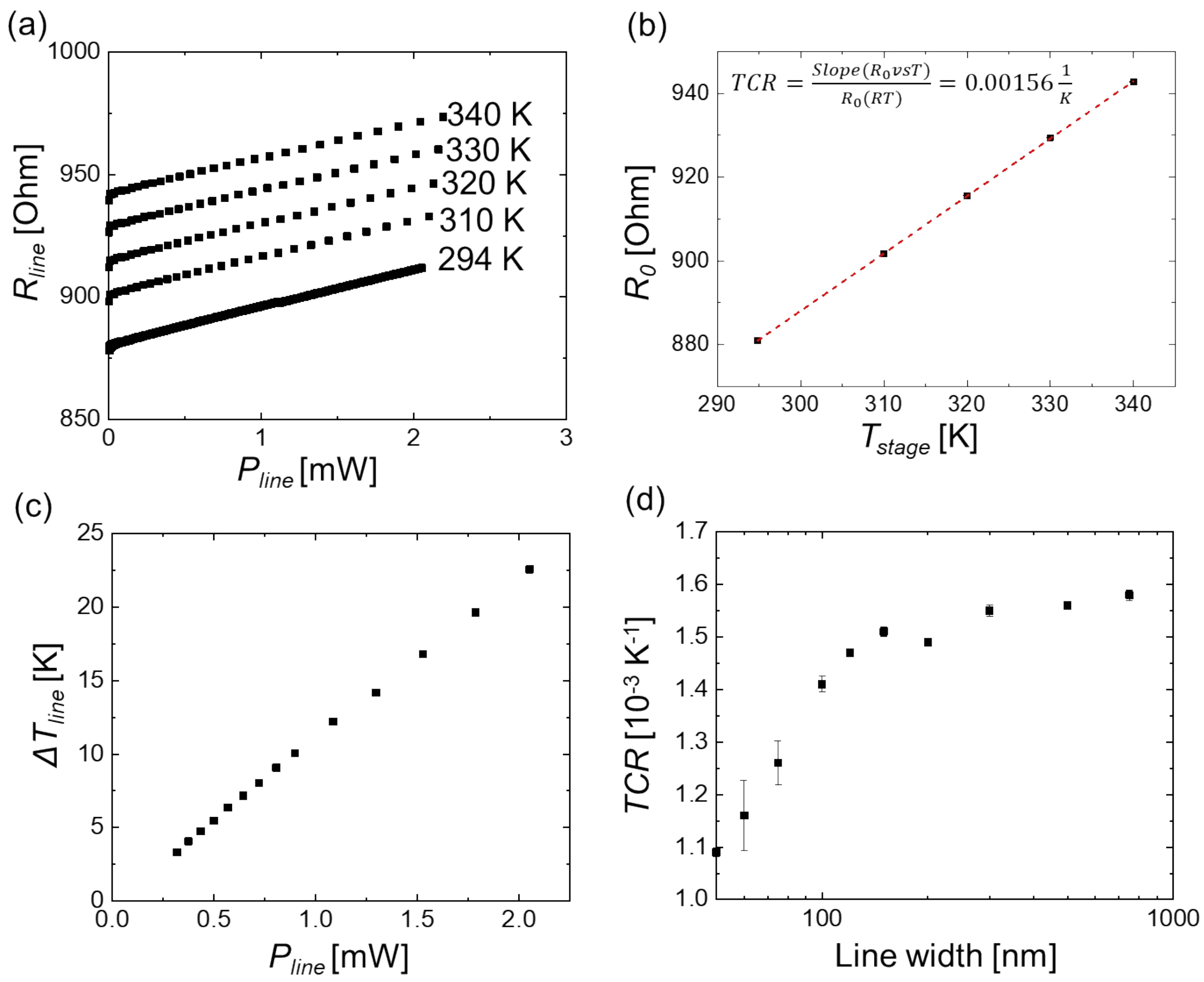
(a)
1000
950
900
850
0
1
2
3
340 K
330 K
320 K
310 K
294 K
R_{line} [Ohm]
P_{line} [mW]
(b)
$TCR = \frac{Slope(R_0 vsT)}{R_0(RT)} = 0.00156\frac{1}{K}$
940
920
900
880
290
300
310
320
330
340
R_0 [Ohm]
T_{stage} [K]
(c)
25
20
15
10
5
0
0.0
0.5
1.0
1.5
2.0
ΔT_{line} [K]
P_{line} [mW]
(d)
1.7
1.6
1.5
1.4
1.3
1.2
1.1
1.0
100
1000
TCR [10^{-3} K^{-1}]
Line width [nm]

Figure S4

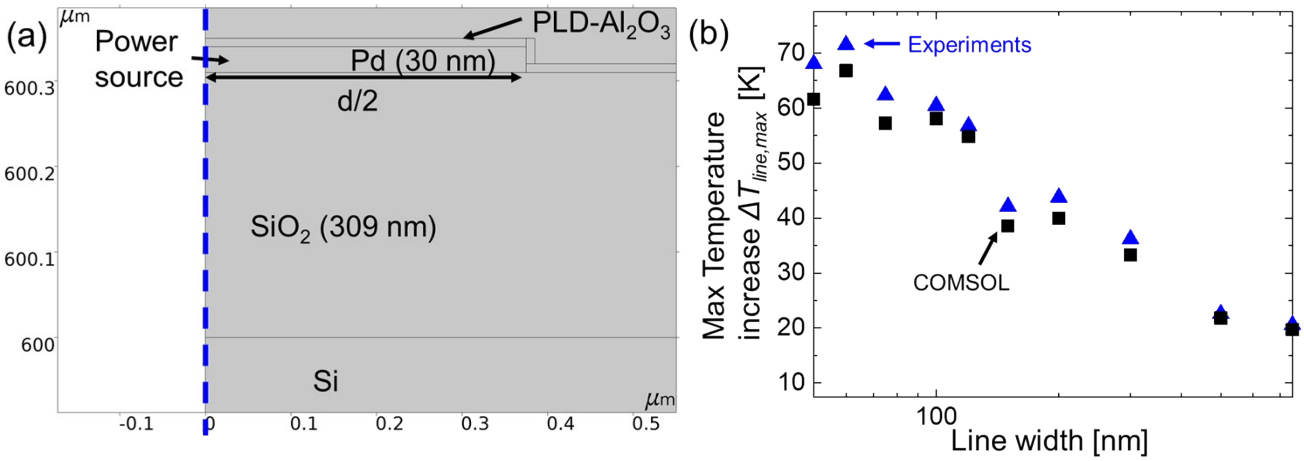
(a)
μm
Power
source
PLD-Al2O3
Pd (30 nm)
d/2
SiO2 (309 nm)
Si
600.3
600.2
600.1
600
-0.1
0
0.1
0.2
0.3
0.4
0.5
μm
(b)
Experiments
COMSOL
Max Temperature increase ΔT line,max [K]
70
60
50
40
30
20
10
100
Line width [nm]

Figure S5

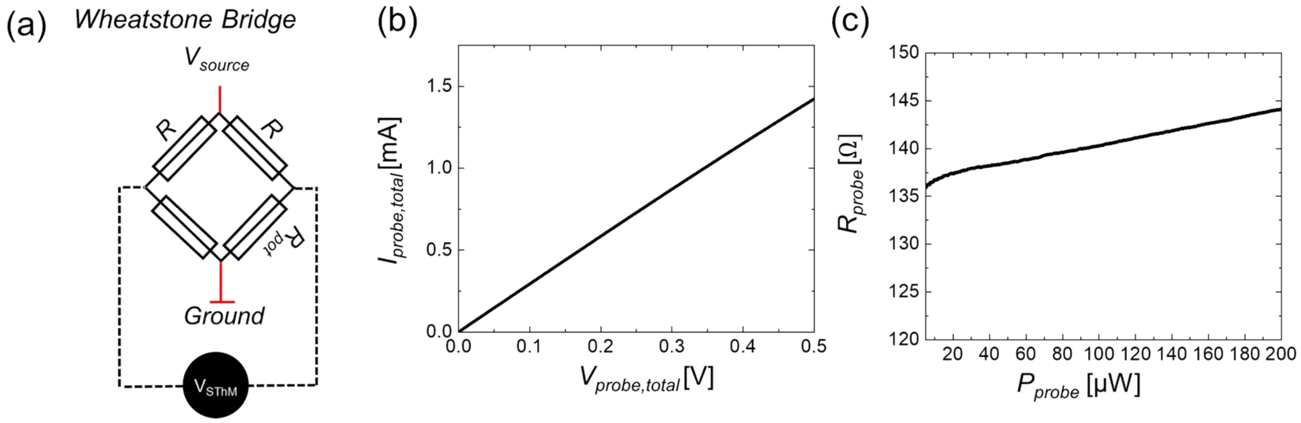

(a)
Wheatstone Bridge
V_source
R
R
R_pot
Ground
V_SThM
(b)
I_probe,total [mA]
V_probe,total [V]
0.0
0.5
1.0
1.5
0.0
0.1
0.2
0.3
0.4
0.5
(c)
R_probe [Ω]
P_probe [μW]
120
125
130
135
140
145
150
20
40
60
80
100
120
140
160
180
200

Figure S6

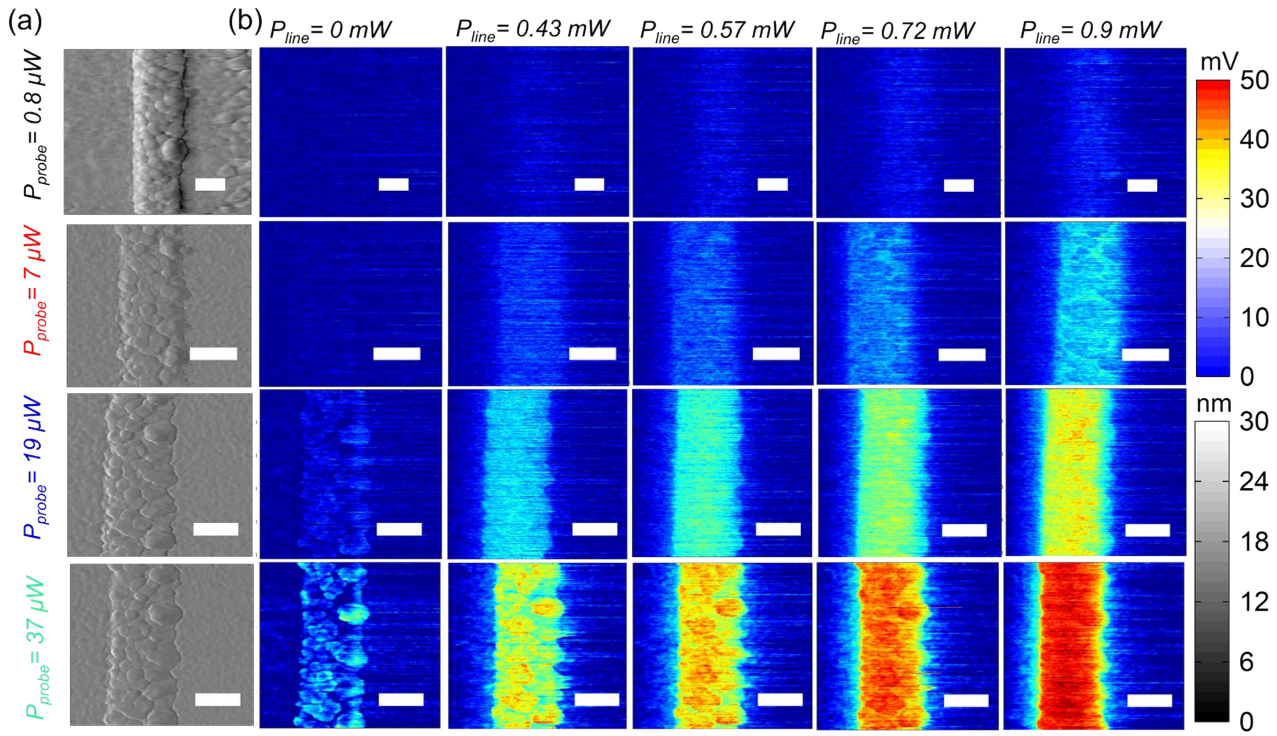

(a)
(b)
P_{line}= 0 mW
P_{line}= 0.43 mW
P_{line}= 0.57 mW
P_{line}= 0.72 mW
P_{line}= 0.9 mW
P_{probe}= 0.8 μW
P_{probe}= 7 μW
P_{probe}= 19 μW
P_{probe}= 37 μW
mV
50
40
30
20
10
0
nm
30
24
18
12
6
0

Figure S7

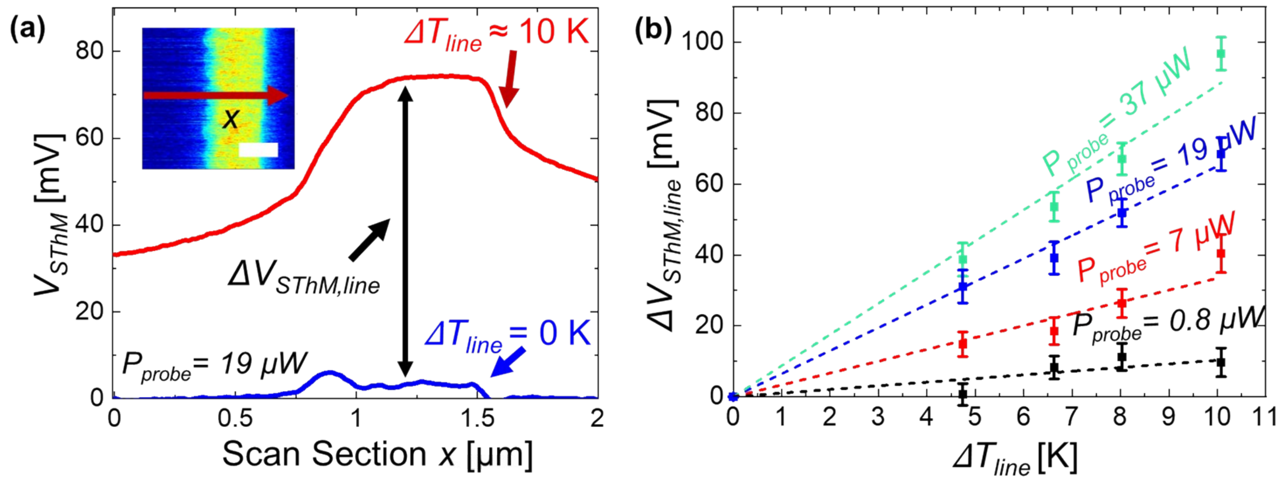

(a)
ΔT_line ≈ 10 K
x
V_SThM [mV]
ΔV_SThM,line
ΔT_line = 0 K
P_probe= 19 μW
Scan Section x [μm]
(b)
ΔV_SThM,line [mV]
P_probe= 37 μW
P_probe= 19 μW
P_probe= 7 μW
P_probe= 0.8 μW
ΔT_line [K]

Figure S8

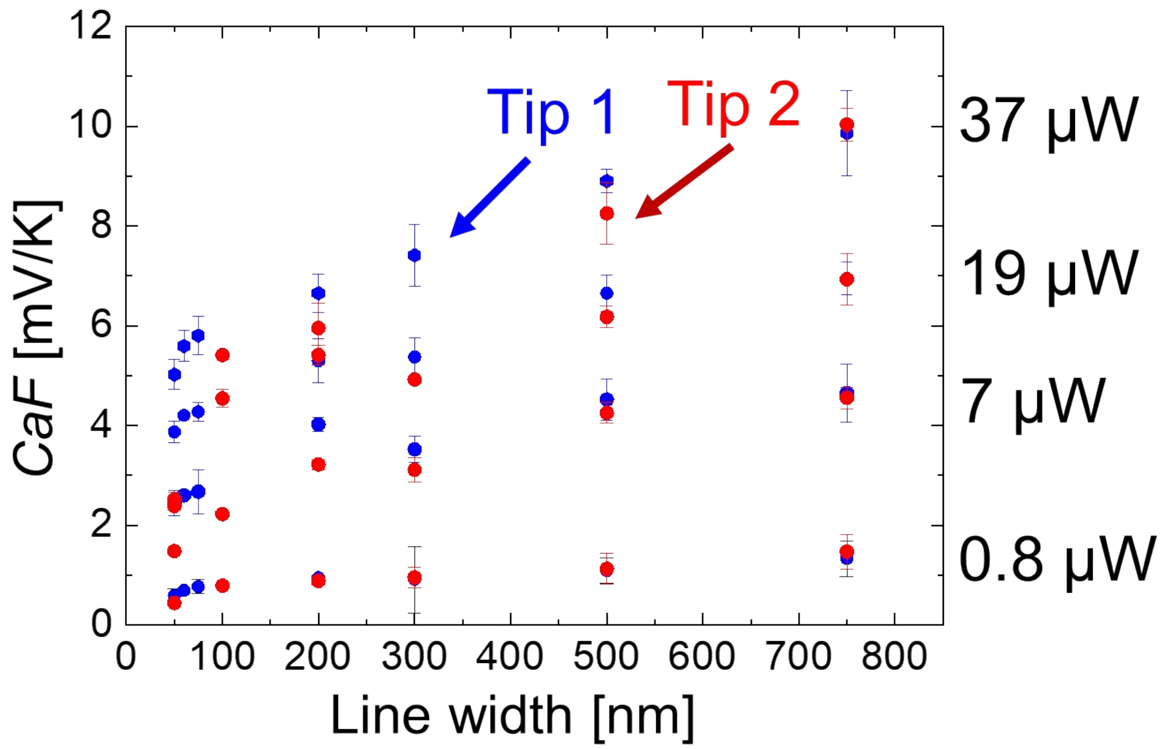

12
10
8
6
4
2
0
CaF [mV/K]
0
100
200
300
400
500
600
700
800
Line width [nm]
Tip 1
Tip 2
37 μW
19 μW
7 μW
0.8 μW

Figure S9

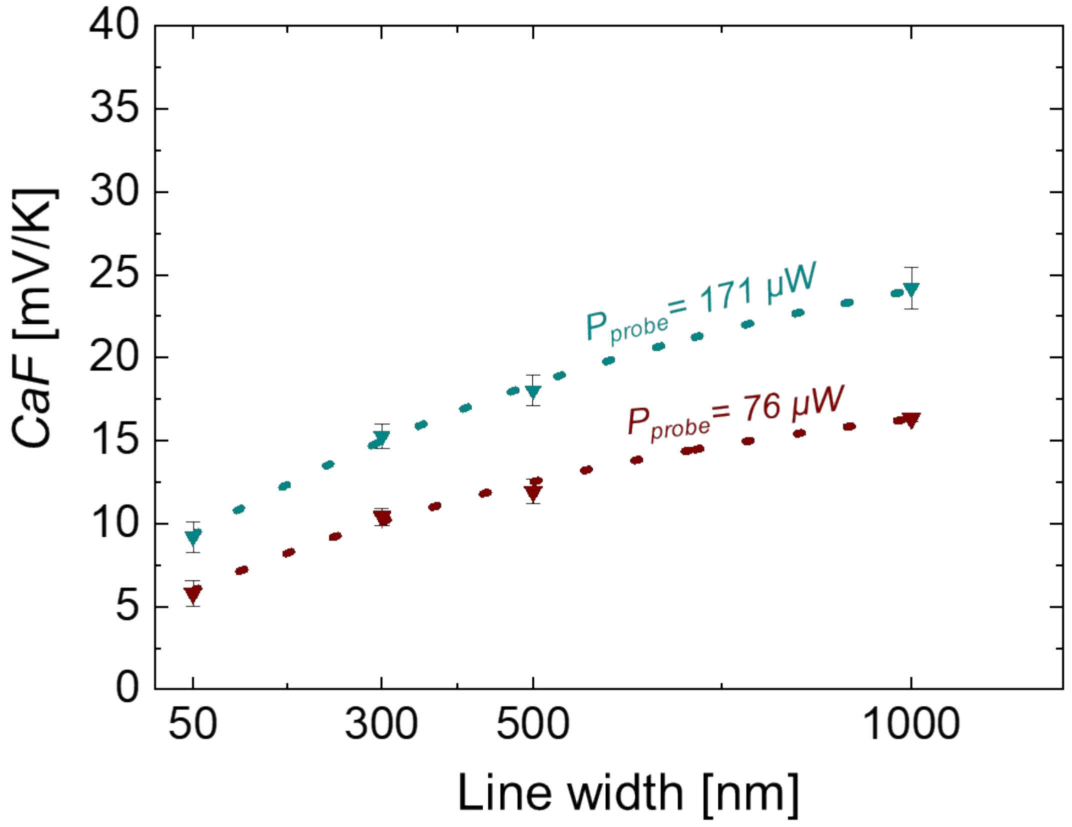

40
35
30
25
20
15
10
5
0
CaF [mV/K]
50
300
500
1000
Line width [nm]
P_probe= 171 μW
P_probe= 76 μW

Figure S10

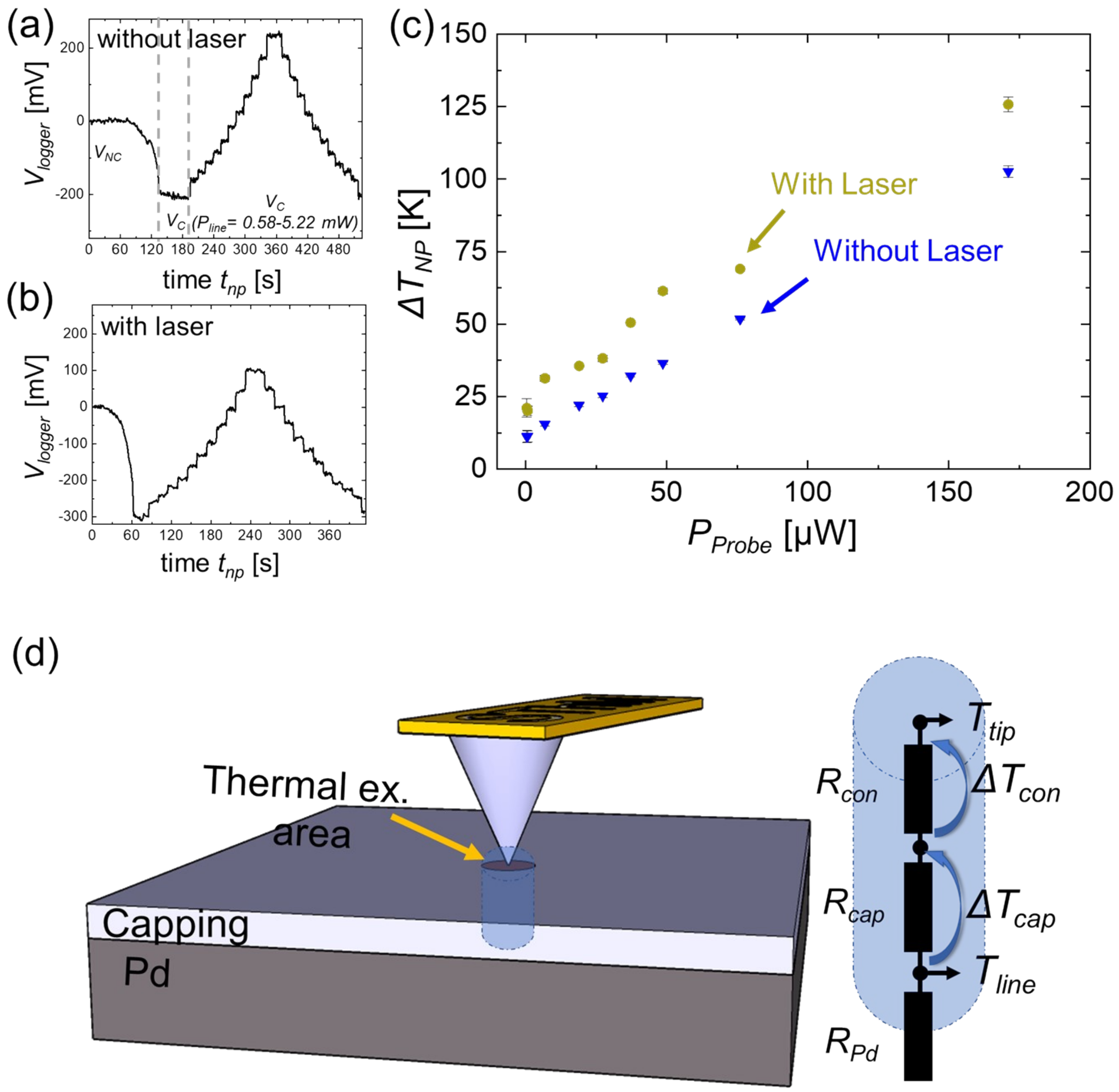

(a)
without laser
V_{logger} [mV]
200
0
-200
V_{NC}
V_C
V_C (P_{line}= 0.58-5.22 mW)
0 60 120 180 240 300 360 420 480
time t_{np} [s]
(b)
with laser
V_{logger} [mV]
200
100
0
-100
-200
-300
0 60 120 180 240 300 360
time t_{np} [s]
(c)
ΔT_{NP} [K]
150
125
100
75
50
25
0
With Laser
Without Laser
0 50 100 150 200
P_{Probe} [μW]
(d)
Thermal ex. area
Capping
Pd
T_{tip}
R_{con}
ΔT_{con}
R_{cap}
ΔT_{cap}
T_{line}
R_{Pd}
