# Supplementary material for: Nanoscale temperature sensing of electronic devices with calibrated scanning thermal microscopy
Source: Nanoscale. 2023 Mar 23;15(15):7139–46. doi: 10.1039/d3nr00343d (PMC10099078; doi:10.1039/d3nr00343d)
Supplement: NR-015-D3NR00343D-s002 [file NR-015-D3NR00343D-s002.pdf]

Electronic Supplementary Material (ESI) for Nanoscale.
This journal is © The Royal Society of Chemistry 2023

Supporting Information: Nanoscale temperature sensing of electronic devices with calibrated scanning thermal microscopy

Timm Swoboda,[a] *Nicolás Wainstein,*[b] *Sanchit Deshmukh,*[c] *Çağıl Köroğlu*,[c] *Xing Gao*,[d] *Mario Lanza,*[e] *Hans Hilgenkamp*,[d] *Eric Pop*,[c] *Eilam Yalon*[b] and *Miguel Muñoz Rojo*[a,f,*]

[a]Department of Thermal and Fluid Engineering, Faculty of Engineering Technology, University of Twente, Enschede, The Netherlands

[b]Faculty of Electrical Engineering, Technion – Israel Institute of Technology, Haifa, Israel

[c]Department of Electrical Engineering, Stanford University, Stanford, USA

[d]Faculty of Science and Technology and MESA+ Institute for Nanotechnology, University of Twente, Enschede, The Netherlands

[e]Materials Science and Engineering Program Physical Science and Engineering Division King Abdullah University of Science and Technology (KAUST) Thuwal, Saudi Arabia

[f]Instituto de Micro y Nanotecnología, IMN-CNM, CSIC (CEI UAM+CSIC), Madrid, Spain

*corresponding author: m.m.rojo@csic.es

S1 Fabrication of the calibration sample

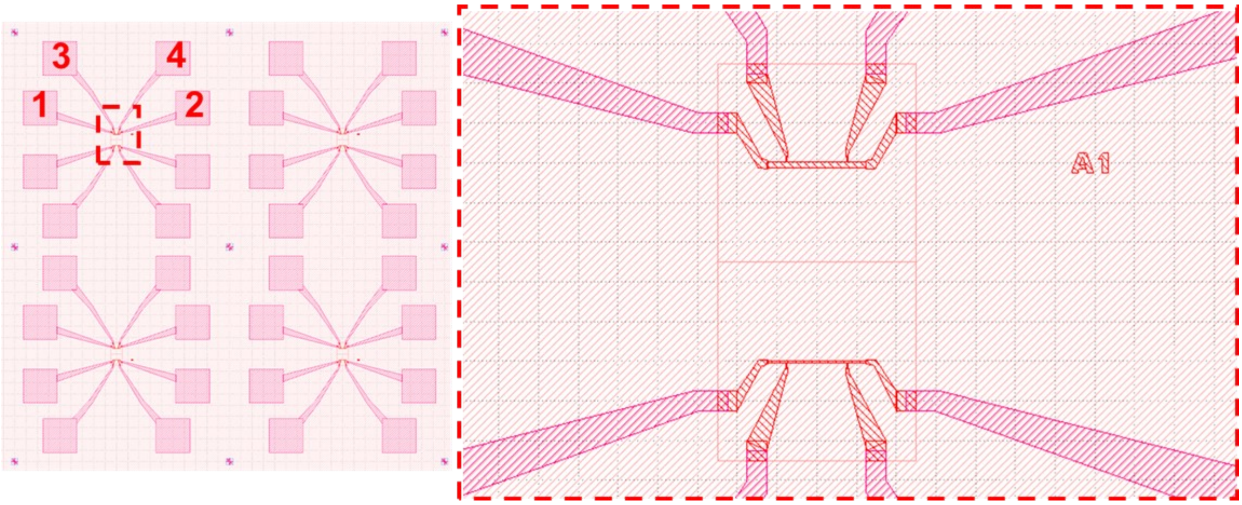

Fig. S1: Mask design of the calibration samples. The heater pads (1-4) are used for four-point probe measurements. The four heater pads are connected via metal lines of a width of 50-750 nm as indicated in the right image.

Firstly, four heater pads were patterned on a SiO_2/Si Substrate by using optical lithography. The substrate was made of boron doped silicon with a thickness of 525 ± 25 µm. The thickness of the SiO_2 was measured to be 309 nm employing an ellipsometer. 2 nm Ti/50 nm Pd contact pads were evaporated through e-beam evaporation. Secondly, 50-750 nm wide heater patterns were fabricated using e-beam lithography using poly-methyl methacrylate (PMMA) as a resist layer. Using e-beam evaporation, we deposited 2 nm Ti/30 nm Pd metal lines. An Al_2O_3 thin film was deposited on these calibration samples using pulsed laser deposition (PLD) from a crystalline Al_2O_3 target at room temperature. The distance between the target and sample was ~ 45 mm. A KrF excimer laser (λ = 248 nm, 20 ns pulse duration) was used with an energy density of ~ 1.5 J/cm^2 and a pulse repetition rate of 5 Hz. The oxygen background pressure was 10^{-1} mbar. All films were grown using the same number of laser pulses (500) and the target thickness was ~10 nm. The mask design is illustrated in Fig. S1.

S2 Atomic force microscopy analysis of the topography of the lines

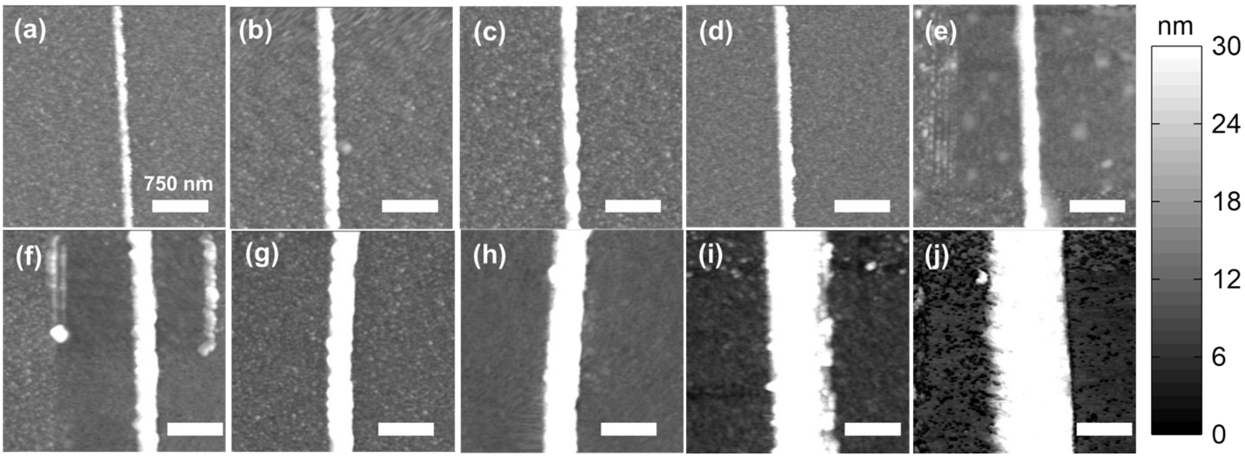

Fig. S2: Atomic force microscopy maps obtained for the Pd metal lines of calibration samples with a line width of **(a)** 50 nm, **(b)** 60 nm, **(c)** 75 nm, **(d)** 100 nm, **(e)** 120 nm, **(f)** 150 nm, **(g)** 200 nm, **(h)** 300nm, **(i)** 500 nm, **(j)** 750 nm (scale bar 750 nm).

We used an Asylum MFP-3D atomic force microscope (AFM) as the tool of all our scans in this study. First, we started scanning the different lines in AFM tapping mode. We scanned the surface by using a constant speed and scan size. **Fig. S2 (a-j)** show the topography obtained for the different lines (all scale bars 750 nm).

S 3. Electrical characterization of the lines

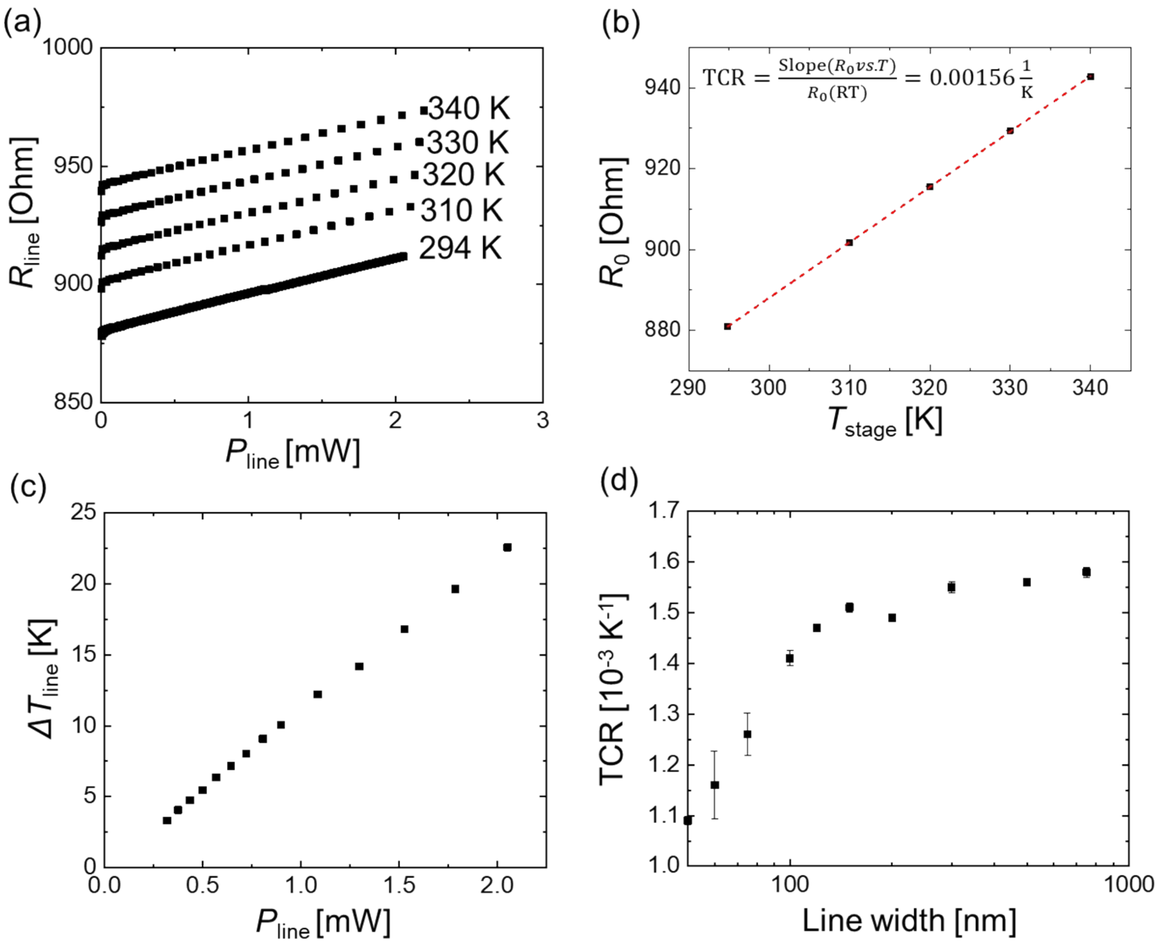

Fig. S3: (a) Resistance of a 500 nm wide Pd metal line R_{line} as a function of the power applied to the line P_{line} obtained at four different temperatures. **(b)** Resistance at zero power R_0 obtained from the results in (a) plotted against the temperature of the sample stage T_{stage} during the measurement. The TCR of the specific line is calculated on base of the slope of this graph. **(c)** Calculated temperature increase of the self-heated line ΔT_{line} plotted against the power applied to it P_{line}. **(d)** Temperature coefficient of resistance (TCR) of each line plotted against their line width.

First, we measured the resistance vs power characteristics of each line. For that purpose, we applied four-point probe measurements to exclude the contact resistance. We applied an electrical current to the outer two heater pads while reading the potential difference at the inner two heater pads. For that purpose, we used a 4200 A-SCS Parameter Analyzer from Keithley. Additionally, we used thin tungsten needles to contact the heater pads. We adjusted the maximum current applied to the lines depending on the line width. We aimed to not exceed a power value of 3 mW to avoid any heating damage to the lines. Moreover, these power magnitudes enabled a considerable elevated temperature increase which fulfilled our requirements. Using a sample stage with an adjustable temperature, we repeated this measurement at four different temperatures (310, 320, 330, and 340 K). **Fig. S3** (a) shows an example of the R_{line} vs P_{line} results for a Pd metal line of 500 nm line width. The graph at 294 K represents the non-heated measurement at room temperature. On the base of these graphs, we calculated the resistance at zero power R_0 by linearly extrapolating the R_{line} vs P_{line} graphs at higher power values. Fig. S3 (b) shows R_0 obtained at the five temperature configurations as a function of T_{stage} for the same line width. We extracted the temperature coefficient of resistance (TCR) from the slope of the R_0 vs T_{stage} graph using,

$$TCR = \frac{Slope(R_0\ vs\ T)}{R_0(294\ K)} \tag{S1}$$

We can now calculate the temperature increase of the lines ΔT_{line} using the TCR as follows:

$$\Delta T_{line} = \frac{\frac{R_{line}}{R_0} - 1}{TCR} \tag{S2}$$

Fig. S3 (c) shows the estimated ΔT_{line} as a function of P_{line} obtained on the results in Fig. S3 (a). We conducted these measurements for all of the other line widths. Fig. S3 (d) shows the TCR of the lines as a function of their line width.

S 4. COMSOL simulation for characterization of the temperature increase

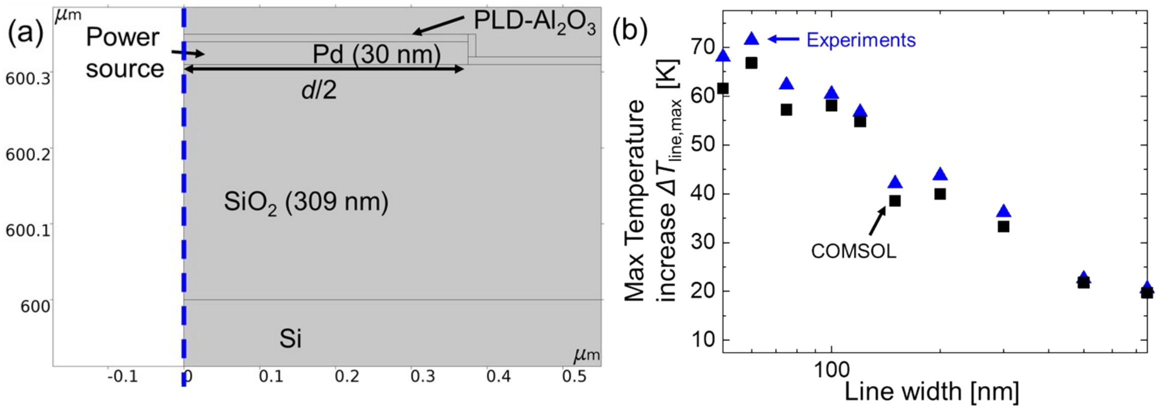

Fig. S4: (a) Geometry of the COMSOL model; **(b)** Max temperature increase $\Delta T_{line,max}$ at the line as a function of its line width, the blue triangles and the black squares represent the results of the 4 Point probe measurements and the COMSOL simulation, respectively. The results were obtained at a power applied to the line of 50 nm P_{line} = 2.45 mW, 60 nm P_{line} = 2.77 mW, 75 nm P_{line} = 2.51 mW, 100 nm P_{line} = 2.72 mW, 120 nm P_{line} = 2.76 mW, 150 nm P_{line} = 2.09 mW, 200 nm P_{line} = 2.42 mW, 300 nm P_{line} = 2.4 mW, 500 nm P_{line} = 2.05 mW, 750 nm P_{line} = 2.35 mW.

We implemented a finite element model (FEM) in COMSOL Multiphysics 5.3 to validate the four-point probe measurements. Fig. **S4 (a)** shows the geometry of the developed model structure. We mimicked the geometry of our calibration sample as described in Fig. S1. For that purpose, we assigned the according material from the COMSOL database to the geometries as illustrated in Fig S4 (a). We choose a lower thermal conductivity for Pd (25 W/(m·K)) and Al_2O_3 (1.5 W/(m·K)) in comparison to their bulk values to take the effect of nanoscale confinement into account. We varied the line width of the Pd (*d)* according to the values of our materials e.g., 750 nm in Fig. S4 (a). Concerning the computation of the results we employed COMSOL's Heat Transfer in Solids module, which solves the heat equation assuming Fourier's law. The ambient temperature was set to 300 K. For the heating of the structure, we applied a heat source to the Pd line geometry. We used a symmetry function on the left edge of the model. Fig. S4 (b) shows the maximum temperature increase of our measurements as a function of the line width. Here the blue triangles and the black squares represent the outcome of the four-point probe measurements and the COMSOL simulation, respectively. Therefore, we extracted the max temperature increase computed at the Pd line while applying the same power density magnitudes as in the measurements of each line indicated in the caption. The results in Fig. **S4 (b)** were estimated at the maximum power values measured during the four-point probe measurements. We concluded that the results of the simulation fit well with the results of the measurements. However, especially at lower line widths the simulated temperature slightly falls below the results of the measurements. This difference results from changes in thermal conductivity at the lower scale as also due to variability of the real line width.

S5. Estimation of the Probe Power during the measurement

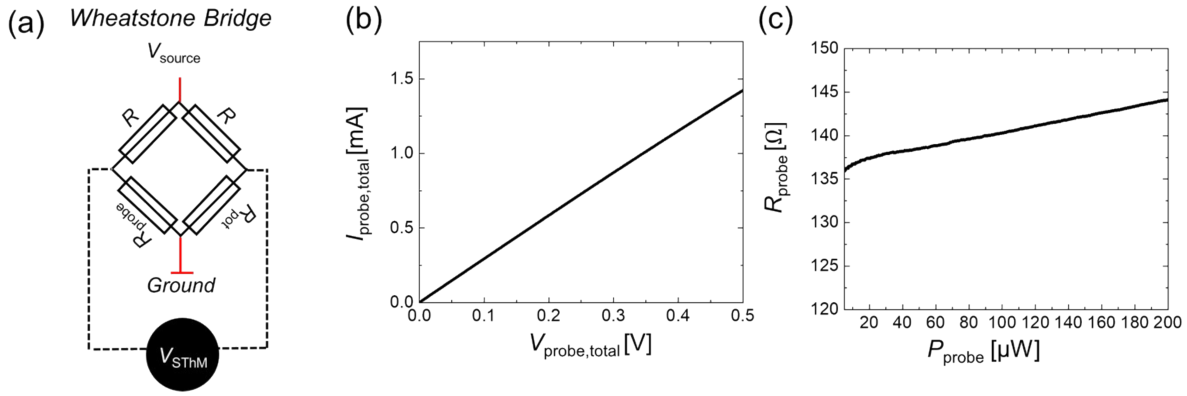

Fig. S5: (a) Schematic of the Wheatstone bridge used for the SThM measurements, **(b)** Electrical current measured at probe $I_{probe,total}$ as a function of the voltage applied to it $V_{probe,total}$ **(c)** Resistance of the SThM probe R_{probe} plotted against the electrical power applied to it P_{probe}.

For the thermal measurements, we used a SThM system from Bruker Anasys connected to our AFM system and thermal probes model GLA-1 from Bruker. These probes consist of a thin Pd resistor on top of a SiN film. The tip radius is around 100 nm. During the measurements, the thermoresistive probe was connected to a Wheatstone bridge as sketched in **Fig. S5 (a).** This electrical network consists of two fixed resistances with R = 1 kΩ, the resistance of the probe R_{probe} and an adjustable potentiometer resistance R_{pot}. To heat the probe, we applied a voltage V_{source} along the bridge to induce a current. To investigate the impact of the V_{source} on the calibration we repeated all the measurements for four different configurations V_{source} = 0.1; 0.3; 0.5; 0.7 V. For all of the four configurations we estimated the power applied to the probe P_{probe}. For that purpose, we measured the resistance of the probe externally by measuring its *R vs P* characteristics with a semiconductor parameter analyzer (SPA). Fig. **S5 (b)** shows the measured electrical current of the whole probe $I_{probe,total}$ as a function of the applied voltage $V_{probe,total}$. Here the probe consists of the actual sensing tip as also two current limiters. We measured the resistance of the current limiters externally to be $R_{current\text{-}limiter}$ = 203.6 Ω in total. The resistance at of the probe R_{probe} was calculated as follows**,**

$$R_{probe} = R_{probe,total} - R_{current-limiter} \tag{S3}$$

We calculated $R_{probe,total}$ by means of the *I vs V* results displayed in Fig. S5 (b). Fig. S5 (c) shows the from equation S3 resulting R_{probe} plotted against the electrical power applied to the probe P_{probe}. From that we can extract the resistance of the probe at zero power to be R_{probe} (0) ≈ 136.6 Ω.

The Wheatstone bridge is a parallel resistance. We calculated the divided voltage at the probe resistance as follows:

$$V_{probe} = V_{source} \cdot \frac{R_{probe}}{R + R_{current-limiter} + R_{probe}} \tag{S4}$$

Subsequently we estimated the power of the probe by using the following equation:

$$P_{probe} = \frac{V_{probe}^{2}}{R_{probe}} \tag{S5}$$

As a consequence, we obtained the power values of the probe P_{probe} = 0.8; 7; 19; 37 μW for the four V_{source} configurations. As can be seen from Fig. S5. (c) the changes of R_{probe} are moderate in the range of applied power. Hence, we can expect that the impact of the temperature dependency of R_{probe} on P_{probe} < 1%.

S6. Scanning thermal microscopy (SThM) measurements

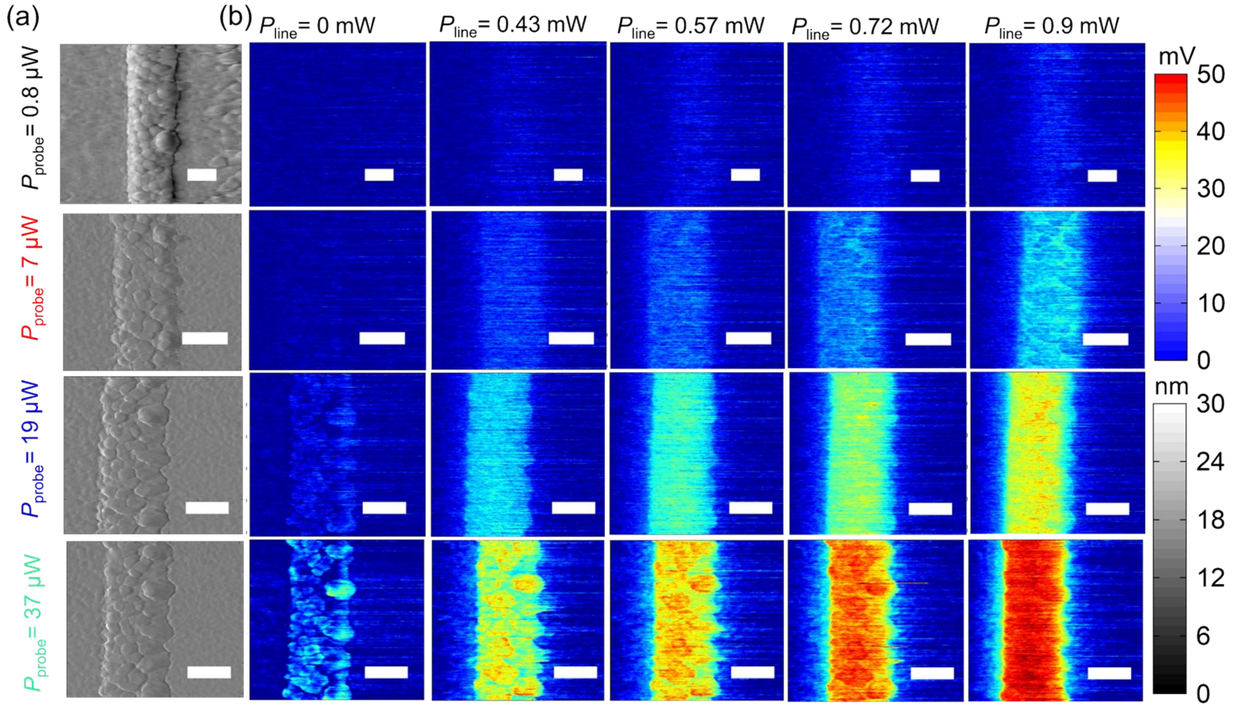

Fig. S6: (a) Flattened topography images obtained on a 500 nm wide Pd metal line for the four different P_{probe} configurations, **(b)** Flattened SThM thermal maps created by means of SThM on a 500 nm wide Pd line at different power values applied to it, P_{line} , for the four configurations of P_{probe}. (scale bar 500 nm).

For the thermal characterization, we adjusted the potentiometer of the biased Wheatstone bridge in order that $R_{pot} \approx R_{probe}$ after bringing the probe in contact with the sample surface. Subsequently we scanned the Pd lines of all line widths with the prior described SThM probes connected to the nullified Wheatstone bridge. **Fig. S6 (a)** shows the flattened topography images of a 500 nm wide line obtained at the four power configurations. We flattened the images by subtracting the average value from each line. During the scans we applied an electrical current along the metal lines to heat the line as explained in S3. We used the SPA and the probe station of our AFM system to induce the current. **Fig. S6 (b)** shows flattened SThM thermal maps obtained on the same 500 nm wide line as in (a) at zero power, and at four different power magnitudes applied to the lines during measurements.

The SThM probe heats up once it scans over a feature of higher temperature in this case the heated line, increasing its electrical resistance. Hence, we used the SThM probe as a thermal sensor that correlates a temperature increase with an increment of the SThM signal across the bridge induced due to changes in the probe resistances. As a consequence, the SThM signal obtained in Fig. S6 (b) at the line increases with the power applied to the line. Additionally, we observed that the SThM signal sensitivity improved as a function of the power applied to the probe P_{probe}. We flattened the images so that the left and right edges approximate a signal change of zero. However, by that, remaining heat at the edges is excluded. Hence, we used the raw data to determine the calibration factor as explained in the following section. It is worth mentioning that we observed significant changes in the line signal at zero power at higher P_{probe} values. At the increased power the probe started to heat significantly. Therefore, heat dissipated from the probe to the surface. The magnitude of heat dissipation depends on the thermal resistance of the material resulting in a difference of the signal from the line to the surrounding. To take that into account we calculated the signal difference from the heated vs non-heated case at the line as explained below.

S 7. Conversion of SThM signal into temperature signal

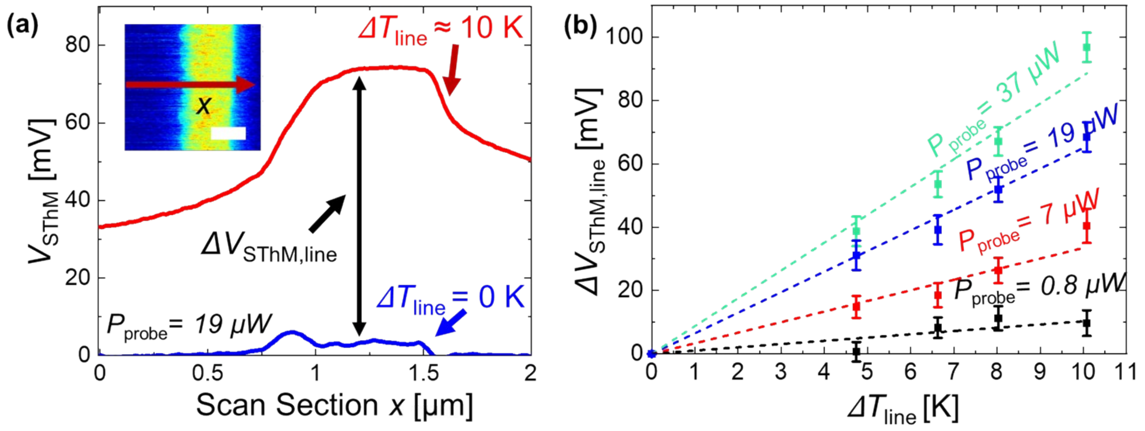

Fig. S7: (a) SThM raw data thermal signal V_{SThM} of a 500 nm wide line obtained at a power applied to the probe P_{probe} of 19 μW obtained across the line section *x* (Scale bar 500 nm in inset figure. The graphs are obtained while scanning over a non-heated line (blue) and a heated line with a temperature increase of $\Delta T_{line} \approx 10$ K (red). **(b)** SThM signal difference $\Delta V_{SThM,line}$ as a function of the temperature of the line for the four probe configurations at a 500 nm wide line. $\Delta V_{SThM,line}$ is obtained by calculating the difference in the V_{SThM} at the line between the heated vs non-heated case, as illustrated in (a).

For the estimation of the calibration factor, we calculated the SThM signal difference between the heated maps and a reference map at zero power. Therefore, we used the raw SThM signal at the line to take the overall temperature increase of the maps into account. Fig. S7 (a) shows the raw SThM signal V_{SThM} obtained at a 500 nm wide line in a heated (red) vs a non-heated case (blue). The change in the thermal resistance of the SThM probe due to nearby topography features causes artifacts in the form of a non-zero SThM signal on the non-heated metal line. Moreover, the general temperature increase causes an elevated signal at the edges of the heated metal line. To determine SThM signals corresponding to the metal line temperatures with minimal influence of topography, we first calculated the maximum signal of each of the scan line $V_{SThM,max,line,n}(T)$. Then we extracted the mean of each line to consider the variation in between each scan line.

Finally, we calculated $\Delta V_{SThM,line}$ between the heated and non-heated case as described in the manuscript in equation 2. Fig. **S7 (b)** shows $\Delta V_{SThM,line}$ of the 500 nm line width as a function of the temperature of the lines during the scan for the four P_{probe} configurations. Here we can see an increase of the *CaF* calculated as the slope of the $\Delta V_{SThM,line}$ vs ΔT_{line} graphs with P_{probe}. We observed this behavior in the remaining metal line widths, resulting in the differences in the *CaF* vs line width graphs presented in Fig. 3 (e) of the main manuscript.

S8. Comparison of the results with a second probe

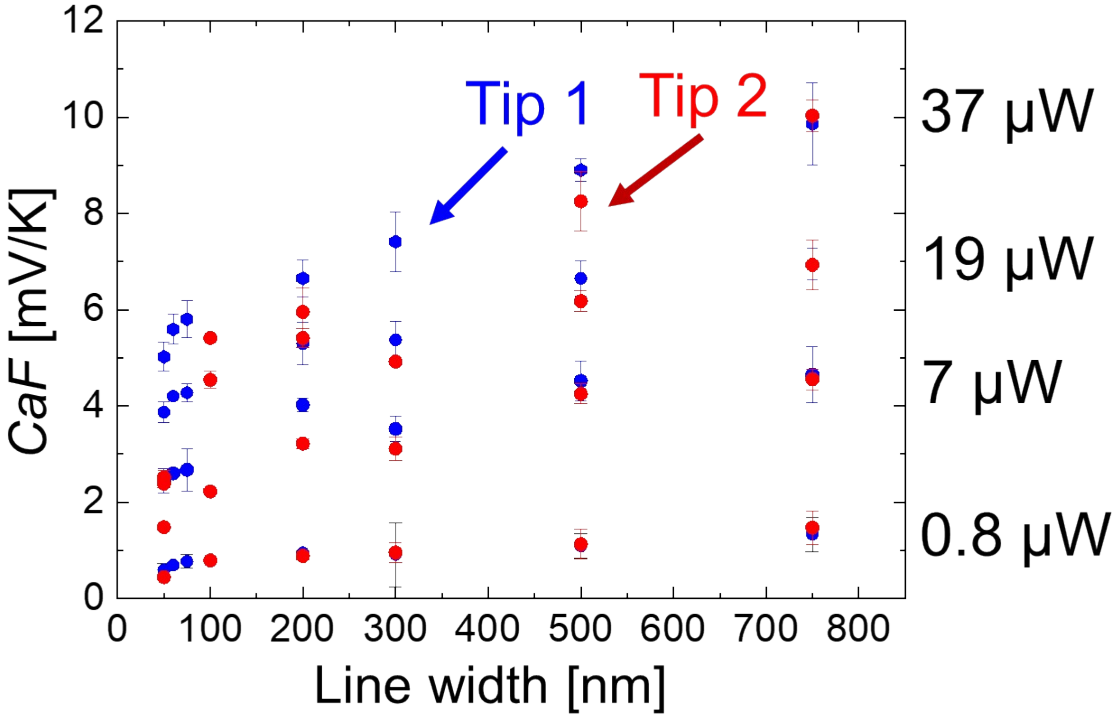

Fig. S8: Calculated calibration factor (*CaF*) for different power values P_{probe} (0.8, 7, 19, 37 µW) as a function of the line width of the scanned metal lines obtained from the retrace signal. The blue dots correspond to the results obtained from the first tip displayed in the main manuscript. The red dots present results obtained on a second reference tip.

We repeated the same measurement procedure as described above for a second probe. **Fig. S8** shows the results of the calibration factor (*CaF)* as a function of the line width of the Pd for both probes. The results of the two tips match well, especially at higher line widths. In both cases, we observed an increase of the temperature sensitivity as well as a shift of the line cut-off width with P_{probe}. At lower line widths we observed a stronger decay of the calibration factor for the second tip. The lower values at the smaller line widths indicate a decreased contact between the tip and the line which might originate from a blunter tip shape of the second probe.

S9. Calibration factor at high power

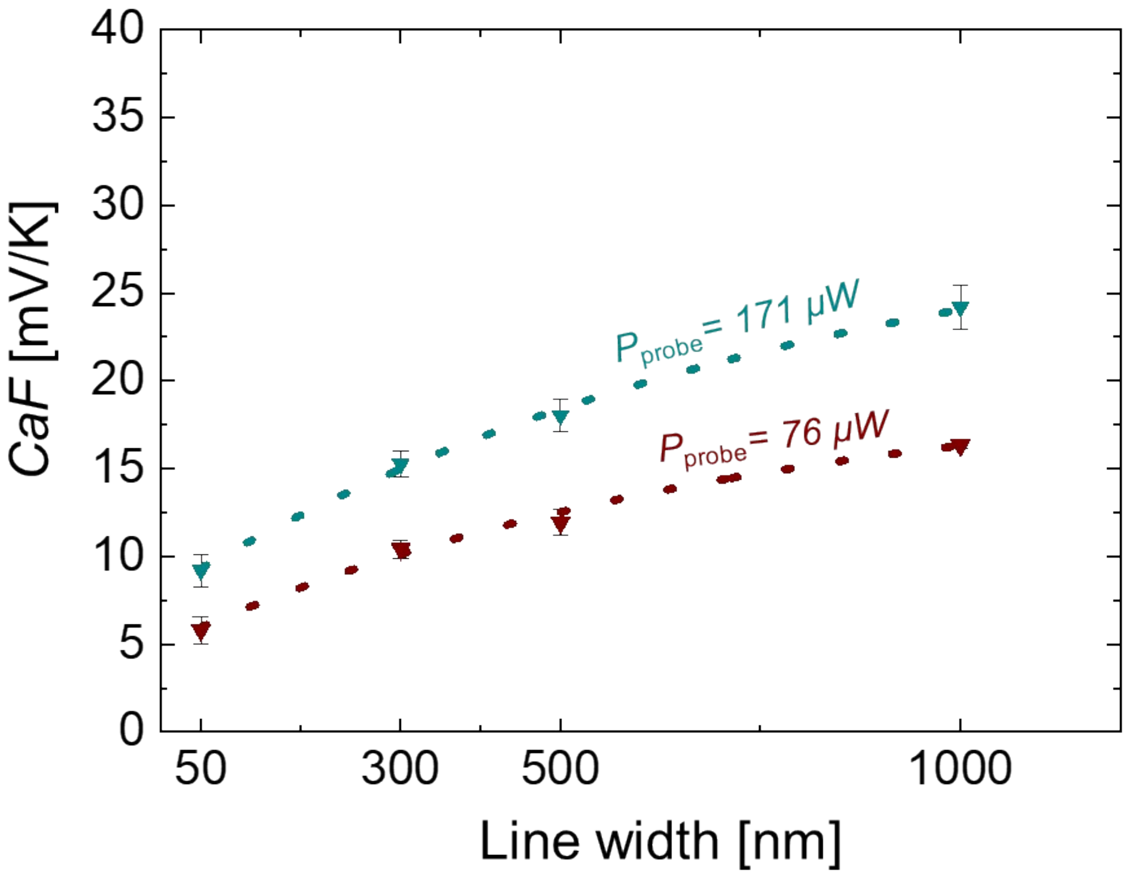

Fig. S9: Calculated calibration factor (*CaF*) for different power values P_{probe} (76, 171 μW) as a function of the line width of the scanned metal lines obtained from the retrace signal. The results are obtained at a Wheatstone bridge voltage V_{source} of 1 V and 1.5 V. The corresponding power values are calculated as demonstrated in section S5.

All the results displayed in the main manuscript are obtained at lower V_{source} values which is common for the operation of the SThM in sensing mode. Fig. S9 shows the *CaF* as a function of the line width obtained at higher power (V_{source} of 1 V and 1.5 V). The results show that the maximum *CaF* further increases with P_{probe}. For application, one must consider that the sample itself heats up significantly at these higher power values which might affect the measuring characteristics significantly. The aim of this study was to characterize the SThM calibration factor at low power values for sensing. If one would like to operate the SThM for sensing at higher power values, additional characterizations would be required. Nevertheless, the results in Fig. S9 show a preliminary trend for these power configurations.

S10. Estimation of the temperature of the probe utilizing Null-Point method (NPM) measurements

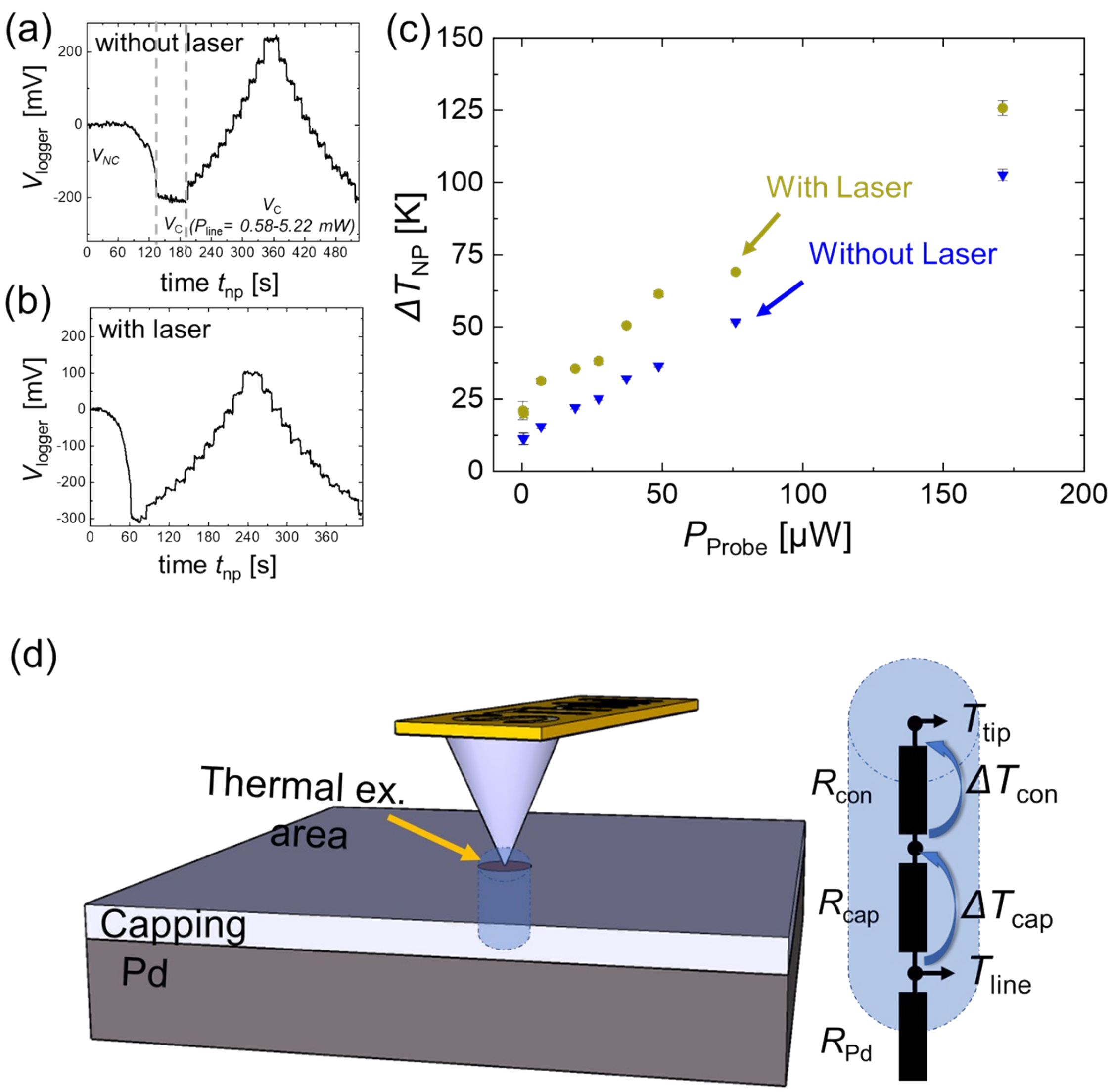

Fig. S10: (a,b) Recorded logger SThM signal V_{logger} of the null-point method estimation as a function of the run time t_{np} while the laser is turned **(a)** off and **(b)** on during the measurements **(c)** Temperature increase of the probe ΔT_{NP} estimated by the null-point method (NPM) as a function of the power applied to the probe P_{probe}. The blue triangles shapes represent the results obtained without AFM laser. The yellow dots show the results with laser during the measurements. The results are obtained at V_{source} = 0.08, 0.1, 0.3, 0.5, 0.6, 0.7, 0.8, 1, 1.5 V. The corresponding power values are calculated as described in section S5 **(d)** Schematic view of the 1D heating model applied to analyze the temperature offset of the NPM measurements.

We applied the null-point method (NPM) to estimate the temperature of the probe as a function of P_{probe}. The NPM is based on the quantification of the probe temperature in contact with the sample T_c *vs* the temperature of the probe in non-contact mode T_{nc}.[1–3] In non-contact mode, the heat transfer between the tip and the sample Q_{ts} is assumed to be 0. Essentially, the temperature of the tip in contact T_c is equal to the temperature of the sample T_S when T_{NC} is equal to T_c.[1] To apply this method to our SThM system we used the logger option of the corresponding SThM software. This option allows to record the SThM signal of the Wheatstone bridge in operando.

Fig. S10 (a,b) show the logger signal V_{logger} as a function of the run time t_{np} without and with AFM laser on during the measurement. First, we tracked the SThM signal in non-contact V_{NC} at which we nullified the signal. Second, we contacted the tip to a 750 nm wide Pd line, lowering the SThM signal V_C as a consequence of the tip to sample heat dissipation. Third, we heated the Pd line steadily to increase the SThM signal again. At one point the SThM signal reached its initial value close to zero i.e., $T_{NC} \approx T_C$ (i.e., $V_{NC} \approx V_C$). Finally, we extracted Tc (ΔT_{NP}) as the temperature of the sample at which this condition becomes true. We repeated these measurements for seven values of P_{probe} and for two cases with the AFM laser on and with the AFM laser off.

Fig. S10 (c) shows the estimated increase of the probe temperature ΔT_{NP} plotted against the power applied to the probe P_{probe} with and without laser. As expected ΔT_{NP} rises linearly with P_{probe} in both cases. Here, we observed a significant difference between ΔT_{NP} with and without laser. The laser heats the probe during the scan up to 25 K more than without the laser. This difference becomes relevant in measurements in which a self-heating of the sample should be avoided. However, the laser is required for the topographic analysis so in most of the cases the measurement with a laser would be the preferred option. In our case, we needed to use the AFM laser to scan the calibration samples, as this is a necessary element for scanning. However, the laser does not affect the relative changes during measurements, as we observed similar slopes in Fig. 10 (c) for both cases. We have measured the samples with the laser on with the effect of having a more elevated background temperature compared to the non-laser case. To reduce the background heating due to laser, one could use different approaches like less laser power of lasers that focus more locally on the tip cantilever. Finally, it is worth noting that ΔT_{NP} does not fall to zero at zero power even without the laser. Here we must mention that the heat transport between the probe and sample is overly complex. Besides the tip-to-sample conduction other parameters must be considered when comparing the non-contact with the contact temperature as heat radiation, thermal contact, or water meniscus.

Fig. S10 (d) shows the schematic of a 1D temperature model, which we applied for the characterization of the impact of the contact resistance on the NPM results. Therefore, we approximated the imaginary power $P_{line} \approx 1.27$ mW (as described in section S3) that we would need to apply to heat the Pd line (line width of 750 nm) to the near zero power temperature of the line $T_{line} \approx 311.2$ K (based on Fig. S10 (c) without laser). By using the COMSOL model described in section S4 we calculated the temperature drop across the capping layer $\Delta T_{cap} \approx 10$ mK. Subsequently we estimated the resistance of the capping layer $R_{cap} \approx 53000$ K/W on base of a cylindrical shape across the capping layer with a radius $r_{thermal\ exchange} \approx 200$ nm approximately equal to the thermal exchange radius of the tip-sample contact as follows:

$$R_{cap} = \frac{x}{k \cdot A} \tag{S6}$$

The thickness $x = 10$ nm, thermal conductivity $k = 1.5$ W/(m·K) and the area $A = \boldsymbol{\pi} \cdot r_{thermal\ exchange}^2$ of the capping layer is equal to the values of the simulation. Based on that we can calculate the heating power across the capping layer $P_{cap} = 0.18$ µW as follows:

$$P_{cap} = \frac{\Delta T_{cap}}{R_{cap}} \tag{S7}$$

We then considered an equal power across the thermal exchange resistance between probe tip and sample R_{con} of the same thermal exchange area. From the literature we estimated R_{con} to have a magnitude of around $4 \cdot 10^6$ K/W.[4,5] By means of that we calculated the estimated temperature drop across thermal exchange resistance ΔT_{con} in this way:

$$\Delta T_{con} = P_c \cdot R_{con} \tag{S8}$$

By using R_{con} from the literature we obtained a ΔT_{con} of 0.75 K. By assuming a thermal exchange resistance of one order of magnitude higher than in literature we would obtain a ΔT_{con} of 7.5 K. Considering the large error of ΔT_{NP} at low power we would obtain the tip temperature T_{tip} to be close to 0, due to the temperature drop across the thermal exchange resistance. According to our estimated ΔT_{NP} offset close to zero power, R_{con} should be estimated within a range of $4 \cdot 10^6$ and $4 \cdot 10^7$ K/W. This result is reasonable as R_{con} can vary significantly in between probes. Additional differences are based on the other heat exchange mechanisms between tip and sample as water meniscus or heat radiation.

References

1 J. Chung, K. Kim, G. Hwang, O. Kwon, Y. K. Choi and J. S. Lee, *Int. J. Therm. Sci.*, 2012, **62**, 109–113.

2 G. Hwang, J. Chung and O. Kwon, *Rev. Sci. Instrum.*, 2014, **85**, 114901.

3 D. G. Cahill, W. K. Ford, K. E. Goodson, G. D. Mahan, A. Majumdar, H. J. Maris, R. Merlin and S. R. Phillpot, *J. Appl. Phys.*, 2003, **93**, 793–818.

4 E. Puyoo, S. Grauby, J. M. Rampnoux, E. Rouvìre and S. Dilhaire, *J. Appl. Phys.*, 2011, **109**, 024302.

5 Y. Zhang, W. Zhu, F. Hui, M. Lanza, T. Borca-Tasciuc and M. Muñoz Rojo, *Adv. Funct. Mater.*, 2020, 30, 1900892.
